# Mortality among the Insane

**Published:** 1919-10-25

**Authors:** 


### MORTALITY AMONG THE INSANE.

One of the subsidiary issues of war conditions has been, according to the recently issued report of the Board of Control, an increased mortality in the mental hospitals. This has resulted, in their opinion, chiefly from the reduction in quality and quantity of the food which it was possible to allow to the inmates of these institutions. As a criticism of this point of view there appeared in the *Times* of September 19 a letter from Dr. T. E. Knowles Stansfield, in which he asseverates that the mortality is not due to the change in food-conditions, but rather to the difficulties in the matter of obtaining adequate nursing supervision. He states that this is the more clearly shown by the fact that the death-rate has been " enormous " among the males as compared with the females because " the mental hospitals were depleted of a very large percentage of their male trained staff," whereas, with regard to the females, " the senior and responsible nursing staff remained practically unaltered by war conditions." In the *Times* of September 24 Sir Robert Armstrong-Jones joins issue with Dr. Stansfield on this matter, maintaining that in the dietary reduction is to be found the solution of the problem.

The truth probably is that both factors have come into play in producing the increase in the mortality rate. It may be stated confidently that the duration of life among many of the insane depends very largely upon the nursing they receive. This is particularly so with bedridden cases, but there are also those who are afflicted by intercurrent conditions, and, in addition, those individuals who, while they do not refuse food to an extent to necessitate the usage of the feeding-tube by the medical officer, take nourishment adequately only when carefully supervised, or have even to be fed by hand by the nurse. Only those who are in constant association with the insane know how much they are dependent on the care and attention of the nursing staff, and it is, very often, the acute and curable cases which suffer most if they do not receive the assiduous ministrations of competent and trained nurses. No amount of nursing, however, can make good a deficiency of diet. Nor can this problem of nutrition among the insane be considered as one and indivisible. It is, it may be repeated, those suffering from acute disorders who need scrupulous care in regard to nutrition, and it should not be forgotten, incidentally, that there have been many who have been nursed through acute attacks of insanity who have done useful work in the world. If the problem is stated in terms of other diseases than those called "mental" the meanest intelligence is able to comprehend the distinction, and can realise that, say, a person who has an attack of acute pneumonia needs more care in every way than another who has a coryza or who is even, like Job, woefully afflicted with boils. As with a bank balance, it is not always sufficient to maintain the balance of nutrition just at the level which suffices for the daily needs. There must be the reserve upon which we can draw to meet a sudden demand.

The increased mortality is, therefore, dependent on various factors. In the first place, it may be due to the restriction of certain articles of diet, then to the vicissitudes as to nursing entailed by war conditions, and finally to the severe epidemic of influenza which found the patients in a condition of diminished resistance. To these may be added, of course, other conditions, such as, for example, tuberculosis, to which the same considerations apply. The higher death-rate among male patients noted by Dr. Stansfield may be explained by saying that, whereas the female patients were subjected only to the restricted dietary, the male patients suffered as a result of this with, in addition, the drawback of inadequate nursing. Incidentally Dr. Stansfield's comment as to the matter of nursing may serve to draw attention once more to the possibility of the employment of women for attendance on the male patients, a method already successfully adopted in some institutions.

